# Supplementary material for: Tropical cyclone-blackout-heatwave compound hazard resilience in a changing climate
Source: Nat Commun. 2022 Jul 30;13:4421. doi: 10.1038/s41467-022-32018-4 (PMC9338923; doi:10.1038/s41467-022-32018-4)
Supplement: Supplementary file 1 — Supplementary Information [file 41467_2022_32018_MOESM1_ESM.pdf]

Supplementary Information for

Tropical cyclone-blackout-heatwave compound hazard resilience in a changing climate

Kairui Feng[a], Min Ouyang[b], Ning Lin[a,1]

[a]Civil and Environmental Engineering, Princeton University, USA

[b] School of Artificial Intelligence and Automation,

Huazhong University of Science and Technology, China

[1]To whom correspondence should be addressed. Email: nlin@princeton.edu

This PDF file includes:

Supplementary text
Figures S1 to S10
SI References

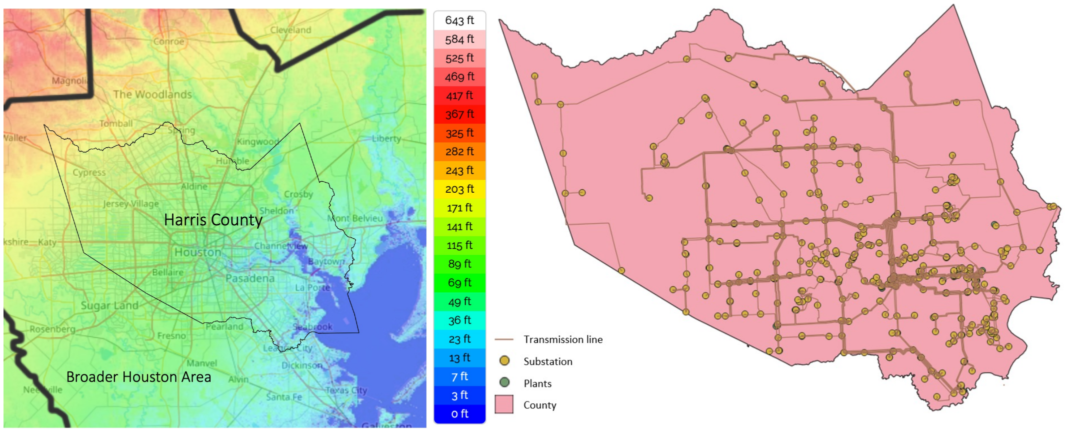

Fig. S1. Geographical representation of the elevation (above sea level; left) and power transmission network (right) in Harris County, Texas. 98% (95%) of the land area of Harris County is over 3 m (6.5 m) above the sea level, and the average elevation is 37 m above the sea level.

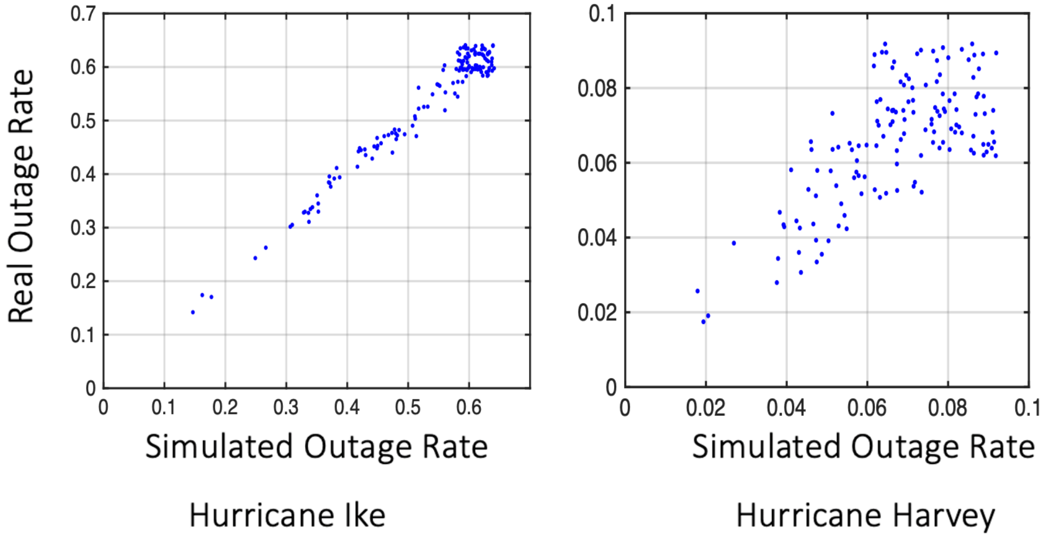

Fig.S2. Comparison of the simulated and observed initial power outage rate for Hurricanes Ike and Harvey at the census tract level (using star-like network topology).

The Mathematical Model for Power Outage Rate. The observed high correlation between power outage rate and the mean length of distribution network and as well as the generalized scaling relationship between the failure probability and failure impact, shown in Fig. 5, can be explained theoretically and generally for acyclic power distribution networks. The acyclic network is the most cost-efficient and usually the only way to design the final-stage distribution networks [1,2]. For the distribution network spreading out from one substation, all branch sections would have equal chance to fail under the same wind impact. Longer branches have more sections and thus are more likely to fail.

The expected power outage ratio (R) of a branch built up with n sectors (with survival possibility of q) could be simply calculated by a hypergeometric distribution (when n and q are large enough):

$$\mathbb{E}(\mathrm{R}|\mathrm{n}) = \sum_{\mathrm{i}=0}^{\mathrm{n}} \frac{n-i}{n} q^{i}(1-q) \approx 1 - \frac{1-q}{nq} \tag{1}$$

After averaging over all the branch sectors and adopting the operator swapping skill tested in ref. [3] and asymptotic expansion when the number of sectors, n, is large and log-logistically distributed, we obtain the log-scaling relationship between the average power outage ratio and the harmonic mean length of the branches (assuming population distribution along each branch is uniform):

$$\mathbb{E}\mathrm{R} \sim \mathbb{E}\big(\mathbb{E}(\mathrm{R}|\mathrm{n})\big) \approx \log\left(\mathbb{E}e^{1-\frac{1-q}{nq}}\right) \sim \log\left(\mathbb{E}\frac{1}{n}\right) \tag{2}$$

Combining this finding with the log-logistic distribution (one of the heavy-tail distributions) of the local distribution network branch length (see Fig. S3), the generalized scaling law automatically emerges for power systems under uniformly randomized attacks on their distribution networks. This theoretical result also confirms that the generalized scaling law of power systems under TC impacts comes from the unbalanced length of distribution network branches.

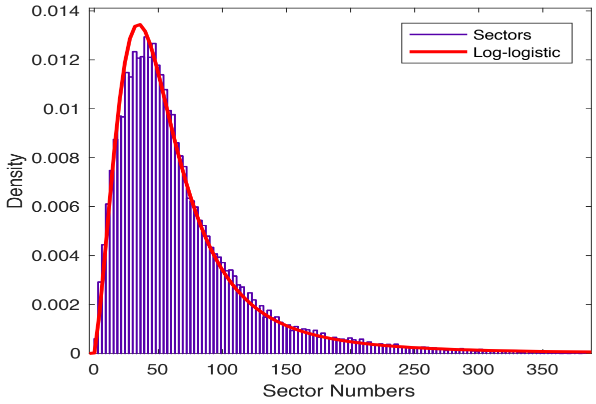

Fig. S3. Length distribution for the star-like distribution network compare to Log-logistic Distribution (the Log-logistic distribution also holds for MST based distribution network)

Risk Analysis for Intermediate Time Slices In this analysis we estimate the evolution of the compound hazard risk over the 21st century. To do so, we linearly interpolate the TC hazard in each GCM to the time slices between historical (1981-2000) and future climates (2081-2100). Then we combine the obtained storm datasets with heatwave data for each time slice for the compound hazard analysis. The obtained results are shown in Fig. S4. For Harris County, the expected percent of customers experiencing at least one longer-than-5-day heatwave without power post-TCs in a 20-year period would increase from 0.8% in the historical climate (1981-2000) to 2.2%, 5.1%, 6.7%, and 18.2% in the

current (2001-2020), near-future (2021-2040), mid-of-the century (2041-2060) and end-of-the century (2081-2100) time frames, respectively.

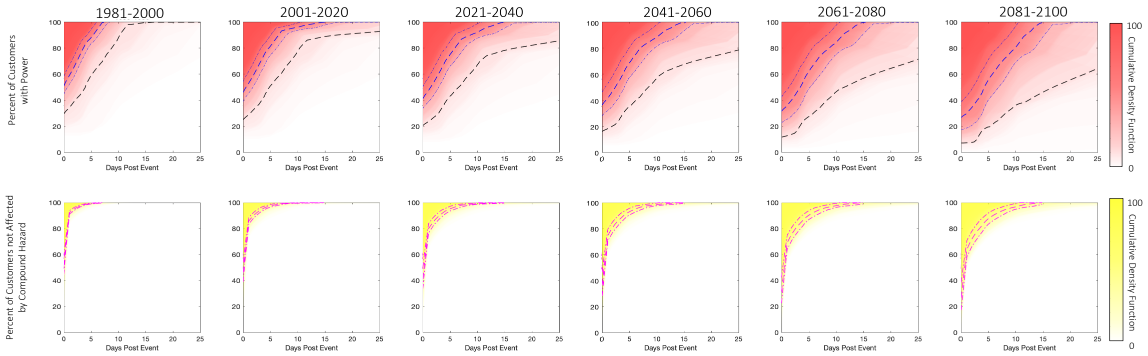

Fig. S4. Same as for Fig. 3 in the main article, except with results added for intermediate time slices.

Discussion about TC Frequency There has been a long debate about how TC frequency will change under climate change, while TCs are predicted robustly to strengthen in a warmer climate. In our model projections, some climate model predicts larger occurrence rates while some give lower occurrence rates. All the model predicts that the average TC intensity will increase. To test the effect of uncertainty in frequency estimation, in this analysis we assume the TC occurrence rate in each climate model remains the same in the future as in the historical period. The resulted compound hazard estimations are shown in Fig. S5. Warming and storm intensification would still significantly increase the compound hazard risk. The expected percent of customers experiencing at least one longer-than-5-day heatwave without power post-TCs in a 20-year period would increase from 0.8% in the historical climate (1981-2000) to 11.2% towards the end of the 21st century (2081-2100).

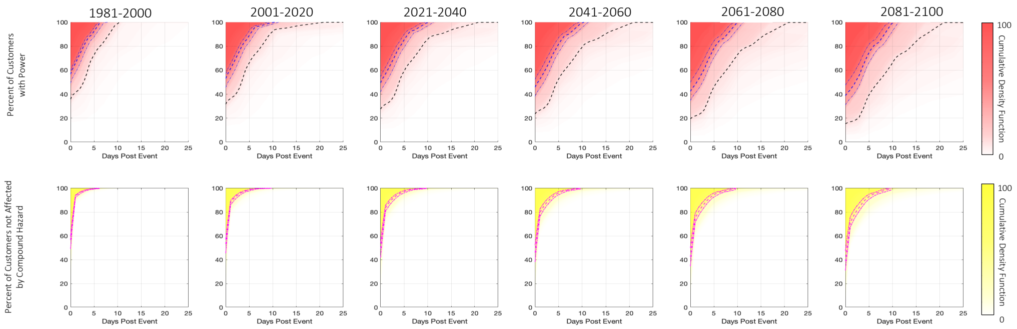

Fig. S5. Same as Fig. S4, except that TC frequency is assumed to remain at the historical level.

RCP2.6 Climate Risk To investigate the lower bound risk, we perform an analysis combining historical TC climatology with RCP 2.6 climate projection for heatwaves. In this analysis the generated TCs and their occurrence probability in the historical climatology remain the same in the future. The temperature and surface humidity time series are taken from CMIP5 RCP2.6 datasets from the 6 climate models. The resulted compound hazard estimations are shown in Fig. S6. In this case, the compound hazard risk changes slightly, with the expected percent of customers experiencing at least one longer-than-5-day heatwave without power post-TCs in a 20-year period would increase from 0.8% in the historical climate (1981-2000) to 1.0% towards the end of the 21st century (2081-2100).

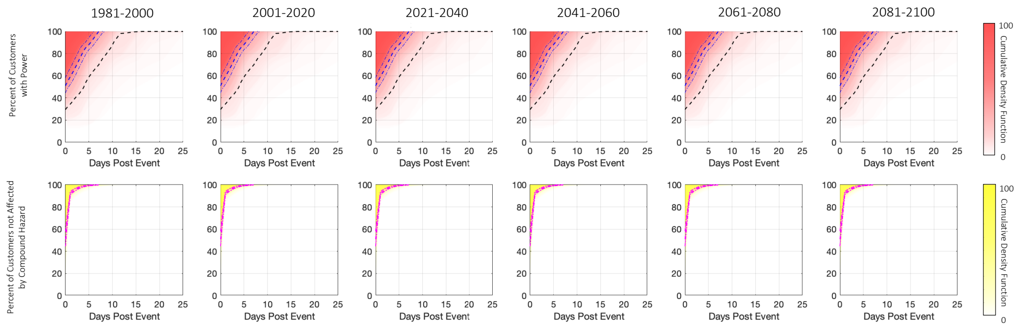

Fig. S6. Same as Fig. S4, except under the scenario that the temperature is projected for the RCP 2.6 emissions scenario and the TC activity (including both frequency and intensity) remains the same as the historical level.

Impact of Heatwaves on Power Restoration Process Without new technology to improve outdoor working condition, outdoor workers may not be able to work efficiently under the extreme heat. Here we simulate the compound hazard risk considering this effect. The Occupational Safety and Health Administration (OSHA) criteria (https://www.osha.gov/heat/heat-index/work-rest-schedules) suggests that "When possible, more frequent shorter periods of exposure to heat are better than fewer longer exposures. This means that the work/rest schedules are often based on 1-hour cycles and might call for a rest period of 15 minutes every hour during hot weather (39.4 - 46.1 degrees Celsius), but 45 minutes per hour when temperature and humidity are extreme (>46.1 degrees Celsius)." We adopt the OSHA criteria in the simulation by tracing the working time and temperature for each worker and restrict every worker's working time according to the criteria. Accordingly, for those workers who work in the daytime and when temperature and humidity are extreme, we shorten their working time. The simulation result is shown in Fig. S7. The expected percent of residents experiencing at least one longer-than-5-day post-TC power outage in a 20-year period is around 53% in the future climate, which is 20% larger than the result ignoring the OSHA criteria (44%). The expected percent of residents experiencing at least one longer-than-5-day TC-blackout-heatwave hazard in a 20-year period is around 23.3% in the future climate, which is 28% larger than the result ignoring the OSHA criteria (18.2%). This analysis confirms that extreme heat can significantly affect the power recovering process.

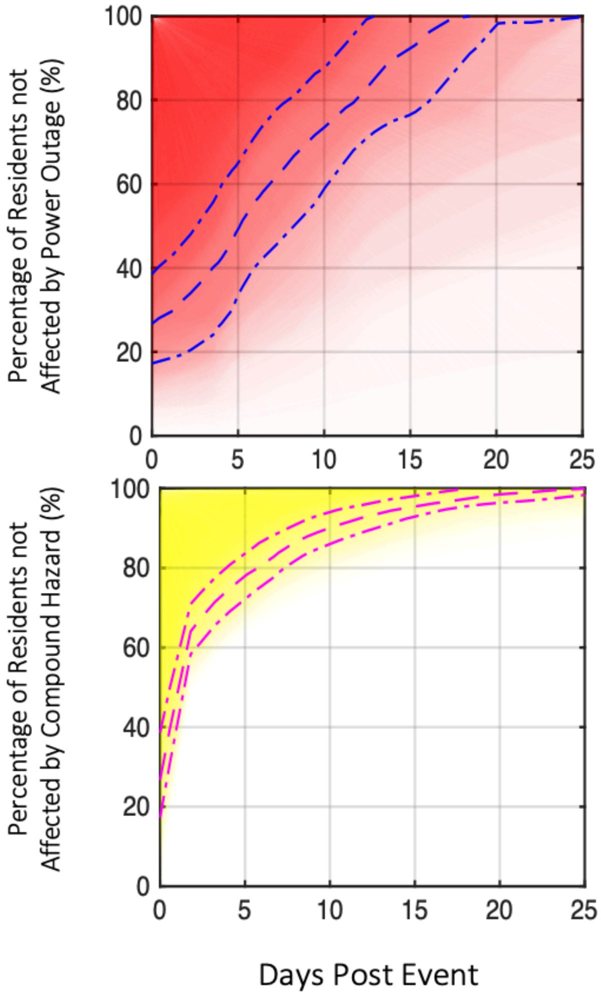

Fig. S7. Same as for Fig. 3 in the main article, except for the OSHA criteria is adopted for the power restoration and only the results for the future climate are shown.

Discussion about Power System Capacity Change There could be a possibility that larger future electricity demand would naturally trigger a capacity upgrade in the power system. Here we are concerning about whether such an upgrade would make the power system more resilient under TCs. In this experiment, we add 10%-50% additional capacity to all the power lines (including transmission lines and distribution lines) and power plants. The obtained results for the future climate (2081-2100) are shown in Fig. S8. Improving the power capacity would not significantly improve the power system reliability and resilience under TCs. The expected percent of customers experiencing at least one longer-than-5-day heatwave without power post-TCs in a 20-year period would decrease from 18.2% to 17.3%, when the capacity is increased by 50%.

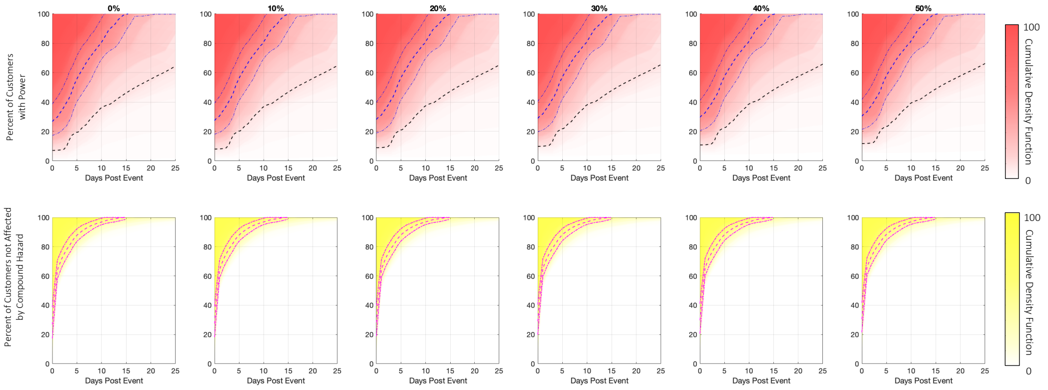

Fig. S8. Same as for Fig. 3 in the main article, except that the capacity of each power line is increased (by the percentage shown above each panel) and only the results for the future climate are shown.

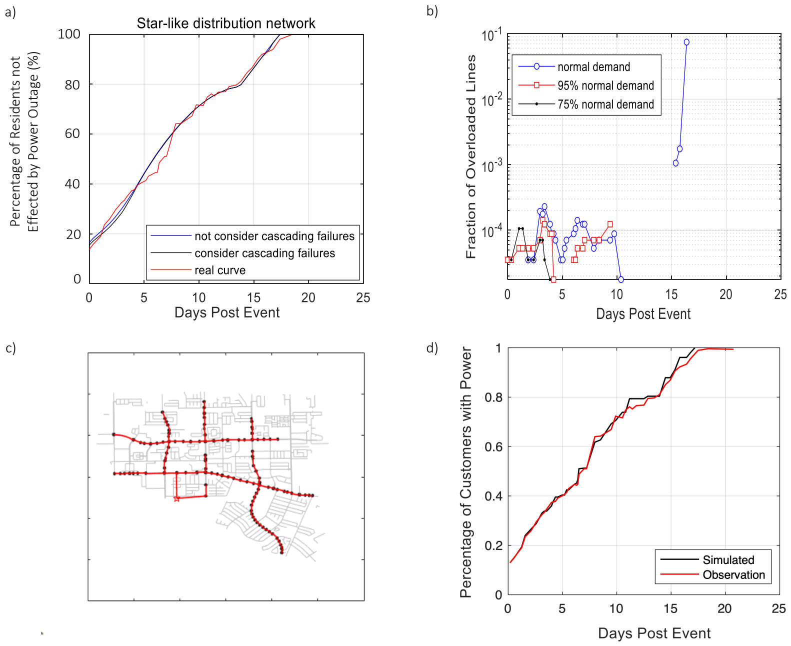

Fig. S9. Impact of assumptions on the distribution network and protective deceives. a) Post-Ike restoration curves averaged over 100 runs in the case of considering cascading failures and not considering cascading failures (i.e., not including protective devices, as they are mainly preventing the power systems from cascading failures), when the distribution networks are modelled as star-like. b) Fraction of overloaded lines during the restoration process when considering the overload-induced cascading failure under normal and reduced power demands (e.g., due to evacuation), for star-like distribution networks. c) Post-Ike restoration curves averaged over 100 runs in the case of considering cascading failures and not considering cascading failures when the distribution network are modelled as MST. d) Post-Ike restoration curves averaged over 100 runs under the

true distribution networks (distribution network topology data obtained from CenterPoint Energy, Inc).

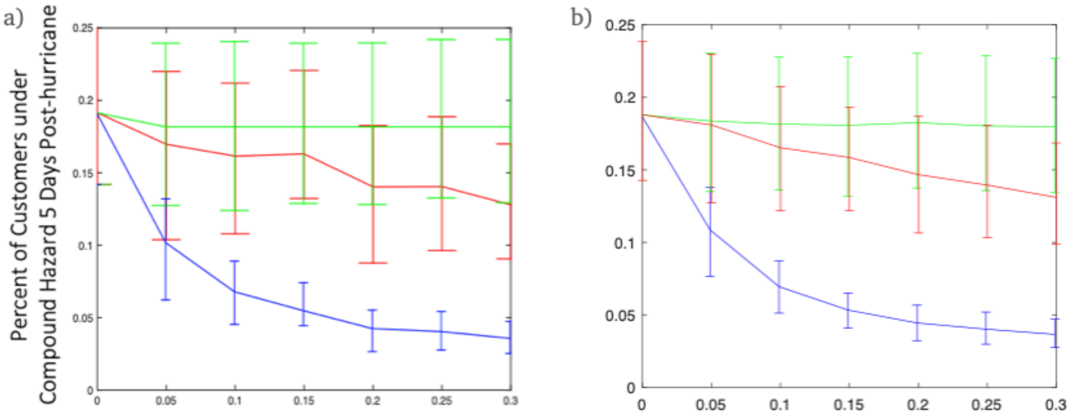

Fig. S10. Same as for Fig. 6, expect for MST-based distribution networks (a) and for the true distribution networks (b).

SI References:

1. Cespedes, R. G. "New method for the analysis of distribution networks." IEEE Transactions on Power Delivery 5.1 (1990): 391-396.

2. Lakervi, Erkki, and Edward J. Holmes. Electricity distribution network design. No. 21. IET, 1995.

3. Gao, Jianxi, Baruch Barzel, and Albert-László Barabási. "Universal resilience patterns in complex networks." Nature 530.7590 (2016): 307.

4. Hines, Paul, Eduardo Cotilla-Sanchez, and Seth Blumsack. "Do topological models provide good information about electricity infrastructure vulnerability?." Chaos: An Interdisciplinary Journal of Nonlinear Science 20.39 (2010): 033122.
